# Adolescents Living with HIV: An Indian Profile

**DOI:** 10.1155/2012/576149

**Published:** 2012-06-24

**Authors:** Sravya Kurapati, Madhu Vajpayee, Meenakshi Raina, Sreenivas Vishnubhatla

**Affiliations:** ^1^Department of Microbiology, All India Institute of Medical Sciences, Ansari Nagar, New Delhi 110029, India; ^2^Department of Biostatistics, All India Institute of Medical Sciences, Ansari Nagar, New Delhi 110029, India

## Abstract

*Purpose*. Although there have been studies on the presence of Human Immunodeficiency Virus (HIV) among the adult and even pediatric population, the adolescent population has been neglected. The main objective of this study was to understand and describe the profile of adolescents accessing the Integrated Counseling and Testing Centre (ICTC) at a tertiary healthcare centre in north India. This was a retrospective analysis of the data collected where, in addition to the analysis of the presence of HIV among the target population, a comparative analysis of HIV-positive and negative individuals was also carried out. *Methods*. Counselors at the ICTC of All India Institute of Medical Sciences recorded responses of the patients, and pre- and posttest counseling was performed appropriately. Also, HIV testing was performed using rapid tests (EIA) and ELISA. Both pre- and posttest counseling was performed for most of the patients. Also, the data collected from 2005 to May, 2011 was then retrospectively analyzed using various statistical tests, such as, Chi-square test and odds ratios. *Results*. Out of 979, 84 tested HIV positive. Discrimination at multiple levels was observed.The 10–14 years age group was 0.56 times more likely to be HIV positive than 15–19 year old. HIV serostatus was strongly associated with risk behavior (*P* = 0.003) with heterosexual transmission being the most common. *Conclusion*. These findings highlight the profile of adolescents in India and their equation with HIV on demographic and psychosocial levels.

## 1. Introduction

An enormous section of the world's population, more than 1.75 billion, is young and aged between 10 and 24 years, making every fifth person in the world an adolescent [1]. Owing to the transitional nature of this age group, they are vulnerable to HIV/AIDS. While HIV prevalence is reducing significantly in many parts of the industrialized world, its decline in the third world countries has been somewhat sporadic, especially in the infective adolescent population. In young countries like India where more than 22.8% [2] of its population is between 10 to 19 years, HIV can be a formidable threat both in terms of incidence and prevalence. Despite the prominence of young adults in the HIV epidemic, prevention research regarding adolescents has been scant. Adolescence is the second decade of life (10–19 years) and is a period of both physical as well as psychological development [3]. This is a phase of experimentation and risk that includes early sexual debut, sexual coercion and violence, trafficking, and substance abuse. Along with these, other factors such as the lack of knowledge about HIV/AIDS, inaccessibility to healthcare services and commodities, lack of education and life skills, and early marriage have increased their vulnerability to HIV/AIDS [4]. Since adolescents comprise a major part of reproductive group, they are likely to play a significant role in determining the future growth pattern of India's population and economy. Thus, it is crucial that investment in terms of finances, research, and developmental policies be done to improve their well being.

The primary drivers of the HIV epidemic in India are unprotected paid sex/commercial sex work (87.1%), unprotected anal sex between men (1.5%) and Intravenous Drug Users (IDUs) (1.6%). Apart from that, 5.4% accounted for parent to child transmission of the infection (PTCT) [5]. Reports of sexual initiation with commercial sex workers vary widely, but a large proportion of unmarried male adolescents and college students report having had sex with commercial sex workers or with older women [6]. Such gender-based sexual patterns are analogous to those observed in other regions of Asia and suggest that young men engaged in unprotected sexual activities could be the bridging population between the high-risk groups (sexual workers) and the general population [7, 8]. Also, these risk behaviors and their prevalence suggest the need for behavior change which could be developed as an effective prevention strategy.

Under the National AIDS Control Program III 2008-09 [9], Voluntary Counseling and Testing Centres (VCTC), and Prevention of Parent to Child Transmission (PPTCT) centres have been remodeled together as Integrated Counseling and Testing Centre (ICTC) and increased to 4987 across the country. These centres provide individual as well as family Counseling, provide the patient with coping strategies against any form of possible discrimination and prepare him/her to lead a safer and healthier life. Thus, they can reduce the frequency of high-risk behavior. The data generated could represent a holistic perspective of the national HIV scenario and as a result could help us understand and assess the situation at hand.

Since India has emerged as a major player in the global HIV epidemic, and given the importance of adolescents in the Indian epidemic, the lack of information on knowledge, perceptions, and behaviors regarding HIV risk and preventive behaviors among Indian adolescents is alarming [10]. In this first study from India, a retrospective analysis was undertaken to describe and understand the profile of adolescents accessing the ICTC services at a tertiary care centre in North India.

## 2. Materials and Methods

This was a retrospective study conducted to analyze the data collected at the ICTC at the All India Institute of Medical Sciences (AIIMSs), a tertiary healthcare centre and National HIV Reference Centre. The data was collected for a period of six years and 4 months from 2005 to May, 2011 and was analyzed in 2012.

### 2.1. Study Population

Unlinked anonymous patient data were collected from the ICTC for the period: 2005 to May, 2011 and specific queries were used to sort out data pertaining to adolescents. Also, all the patients involved in the study were first-time visitors to the ICTC and were usually referred either by clinicians or came to the centre voluntarily. Furthermore, written and informed consent was taken from each patient prior to both their Counseling and testing. In case of minors (<18 years), the details about the test were explained to the parents or any guardian who accompanied them to the centre.

### 2.2. Integrated HIV Counseling and Follow-Up Procedure

All the patients seeking voluntary counseling were interviewed by counselors trained and authorized by the Delhi State AIDS Control Society (DSACS), a state level body of the National AIDS Control Organization (NACO). Pretest counseling was performed for every individual wherein the option to go through a screening test for HIV was explained to the patient and informed consent was obtained. Patient-specific information about personal risks, details about risk behavior, modalities for prevention, HIV testing procedures, their limitations, and the interpretation were discussed at length.

3 mL whole blood was collected from patients for HIV testing. The same counselor interviewed the patient pre- and posttest to maintain patient confidentiality. Posttest counseling included informing the adolescent of his/her HIV test result. In case of a negative or intermediate result, the concept of window period was explained, and they were encouraged to repeat the test after a gap of three months.

In the event of a positive test result, the newly diagnosed adolescent was explained the implications of his/her HIV status. Psychosocial support was provided by preparing and assisting the adolescent with disclosure of his/her HIV status to their family and peers. They were provided with strategies to cope with emotional distress. Apart from this, they were explained the importance of adherence to medication, overall medical care, and their needs associated with emerging sexuality, including mechanisms for communicating HIV status to partners and avoiding high-risk behaviors. Also, the need for prevention of further transmission by heterosexual, homosexual routes, and intravenous drug abuse was stressed upon. In case of sexually active adolescents, HIV test for their partner or spouse was recommended. Counselors equipped patients with management strategies and also provided support to the patient to adjust to his/her HIV status. Apart from support, the patients were explained the importance of monitoring strategies, that are, CD4 counts and viral load that were to be regularly done during follow-up visits in order to monitor their disease and treatment.

### 2.3. HIV Testing

HIV screening as well as supplementary tests were performed in order to establish the HIV status of the patient. These diagnostic tests were performed in accordance with the testing strategies designed and adopted by the National AIDS Control Organization (NACO). For the initial years of the study, ELISA was performed. Nonreactive samples from the first test were declared HIV negative whereas the reactive ones were further tested by Enzyme Immunoassay (EIA). A nonreactive result at this stage was considered negative. But for a reactive result, the sample was retested with a second EIA. Samples reactive on all three tests were considered HIV antibody positive. However, towards the later part of the study duration, three rapid tests each based on a different principle were used to determine the HIV status of the patient.

### 2.4. Measures

Information regarding different variables was recorded after the completion of a questionnaire that is provided each year by DSACS, NACO. As stated above, trained counselors would verbally ask the patient about their demographic details, their familial relationships, their relationships (both sexual and emotional) with their partners, and the type of support they need. Based on the responses provided by the patient to these questions, the questionnaire was filled.

### 2.5. Statistical Analysis

The data was analyzed using Stata, version 11.2.The frequencies of different categories were generated using this software.The odds ratio of HIV positivity for various risk prone behaviors was calculated along with 95% confidence intervals. No adjustment was done for the logistic regression models.

## 3. Results

### 3.1. Sociodemographic Analysis

During the period of 2005 to May 2011, the total patients accessing the ICTC services were 15,220, out of which 1017 were adolescents aged from 10 to 19 years.Most of the patients in this population consented to get tested for HIV. However, 38 refused to undergo the test and were hence excluded from our analysis. Thus, the population that was analyzed comprised of 979 adolescents only. Also, during the analysis of some variables, the sample size marginally decreased due to lack of data. 

**Figure 1:**
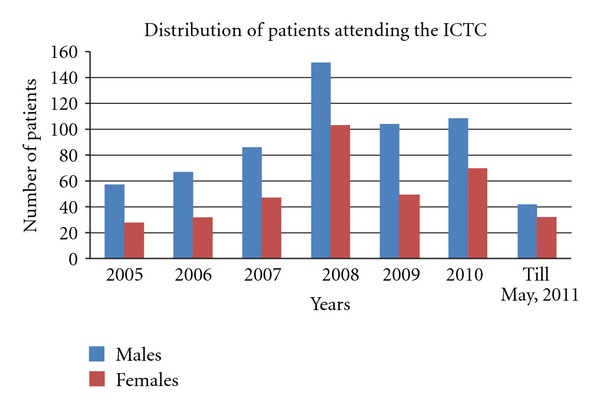
Distribution of participants seeking services at the ICTC across 6 years and 4 months.

Of the 979 adolescents, 615 (62.95%) were males and 362 (37.05%) were females (Figure 1). The median age for this population was found to be 16.24 ± 2.79 years. The study population was categorized into two groups: group I: 10 to 14 years and group II: 15 to 19 years. It was observed that while 233 (25.69%) of the total adolescents belonged to group I, a sizeable 674 (74.31%) constituted group II (Table 1). Also, the difference between males and females in these ages groups was found to be slightly significant (*P* = 0.022).

**Figure 2:**
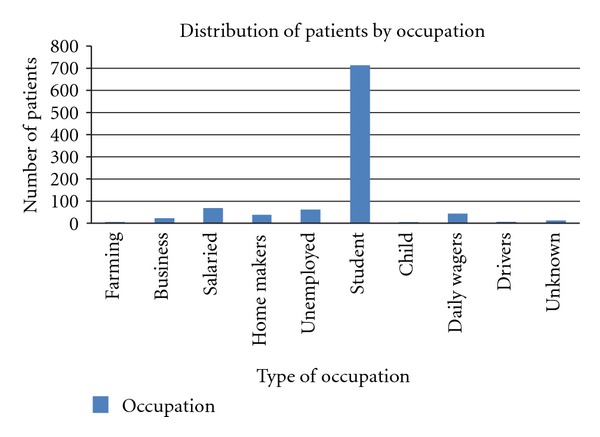
Distribution of the participants on the basis of their Occupation.

While, 8.70% of the population was illiterate, 39.86% of the adolescents was educated till the primary level, 43.27% was educated till the middle and secondary level and the rest (8.18%) were college students. Additionally, the level of illiteracy was seen to be marginally higher in females at 10.61% as compared to males (7.59%). In case of occupation, a majority of the adolescent population (72.82%) was students by profession and the salaried service sector constituted 7.25%. These were followed by homemakers and unemployed adolescents (Figure 2). While greater part of the study population that is, 94.5% was unmarried, 5.5% was married (Table 1). Of this, the proportion of married females was more than males with the difference being highly significant (*P* = 0.001).

### 3.2. Referred by

For the most part of 2006 to 2009, patients voluntarily sought ICT with 60.14% being self referred adolescents. Out of these, 62.70% and 55.73% were adolescent boys and girls, respectively. Furthermore, it was noted that 27.10% was referred to the ICTC by the Direct Observed Therapy (DOT) centre whereas 13.04% was from nongovernmental organizations (NGOs). Data for the remaining years was not recorded and hence was unavailable for analysis.

### 3.3. Risk Behavior

**Figure 3:**
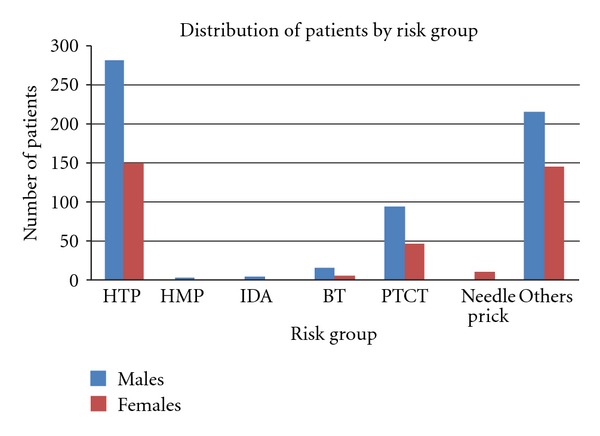
Distribution of the participants on the basis of their Risk Behavior: HTP-heterosexual promiscuous, HMP-Homosexual promiscuous, IDA-Injected Drug Abuse, BT-Blood Transfusion, PCTC-Parent to child transmission, and others.

Each patient was classified by the counselors into the most appropriate risk behavior category based on their responses to the questionnaire used by the counselors. Heterosexual promiscuous (HTP) was the most common risk behavior recorded and accounted for 44.10%. This was followed by PTCT and Blood Transfusion (BT) which made up 14.46% and 2.15% of the study population, respectively (Figure 3). Also, in terms of the risk groups, there was significant difference between males and females (*P* < 0.0001).

### 3.4. Testing for HIV

All the patients included in our analysis had agreed to get tested for HIV, of which 84 had tested positive for HIV antibodies with 67.85% being male and 32.14% female. These were then referred to the Antiretroviral Therapy (ART) Clinic which would assess their clinical condition and decide on the course of action. Also, the suspected TB cases were referred to the Revised National Tuberculosis Control Program (RNTCP) Centre to get screened for TB.

### 3.5. Posttest Counseling Analysis

While the data for posttest counseling was available only for the years 2006–2009, information regarding the same for the remainder years was not noted and thus could not be included in our assessment. However, as recorded for 2006–2009, a significant portion of the study population 94.20% returned for both their reports and counseling. Observations made by the counselors during these sessions showed that 47.97% of the patients shared the outcome of their HIV test with their father followed by 24.92% who disclosed it to their mother. While 11.44% chose to share the result with their spouse, only 6.9% shared it with their partner. An important observation recorded was that 5.36% of the adolescents refused to divulge the result of their test to anybody.

### 3.6. Area of Discrimination, Support Needed

On the basis of the data accumulated from 2006 to 2008, types of discrimination experienced by the patients and the support required by them were recorded. During their interactions with the participants, counselors questioned them whether they felt discriminated against because of their HIV status. Discrimination was perceived by the patients at multiple levels: by family members, by spouse, in financial issues, professionally at work place, schools, and healthcare centres. Based on their assessment of the patient and their psychosocial health, counselors determined the type of support the participant needed.

### 3.7. Association of HIV Serostatus with Level of Education, Occupation, and Risk Behaviors

Of the 979 that were tested for HIV, 84 tested positive for HIV antibodies. Among these, the probability of patients belonging to group I being HIV seropositive was 0.56 times more than those belonging to group II (*P* = 0.001). Also, with respect to HIV seropositivity, the difference between individuals of these two age groups was found to be highly statistically significant (*P* < 0.0001). However, in the case of education as a risk factor, no statistically significant difference was found between those testing positive and negative for HIV. Another variable that was found to be associated with HIV seropositivity was marital status. A significant difference was observed between married and unmarried individuals testing positive for HIV, thereby suggesting a strong association between the two variables. The possible association between HIV seropositivity and marital status especially in case of females was analyzed and found to be highly significant (*P* < 0.0001). On analyzing the risk behaviors recorded, it was noted that a significant difference was recorded between males and females for the different risk groups (*P* < 0.0001). Furthermore, the proportion of HIV-positive males reporting HTP was more (49.12%) as compared to the other risk behaviors. Also, HIV seropositivity was observed to be significantly different for the various risk groups (*P* = 0.003). However, more specifically, in the case of the 10–14 years and 15–19 years age groups, the difference in the HIV serostatus for all the types of risk groups was marginally significant [*P* = 0.034, *P* = 0.014 resp.].

One performing logistic regression, patients exhibiting heterosexual promiscuity were found to be 2.09 times more likely to be HIV positive than those whose risk group could not be determined (*P* = 0.013). Similarly, adolescents classified into the parent to child transmission risk group were more 3.57 times more predisposed to HIV (*P* < 0.0001). Also, adolescents with a history of blood transfusion were 4.80 times more prone to HIV than others (*P* = 0.010).

## 4. Discussion

Ironically, very little work has been done to comprehend the nature of the epidemic in Asia, especially in India which harbors the third highest HIV-affected population in the world. Although the government of India conducts HIV Sentinel Survey annually to monitor outcomes and impacts of national efforts and monitor trends in HIV prevalence amongst the major population groups [11], not much information has been gathered about the adolescent population specifically. Through this retrospective study we have tried to bring to the foreground, the prevailing profile of adolescents living with HIV in India.

One of the major findings of our study was the appalling disparity between the sexes at different levels, their association with HIV and its impact on their daily lives. While most studies have focused on the late adolescents, our study also sheds light on the children aged between 10 and 14 years who often get blanketed under the pediatric population. This transient population is extremely important as not only do they constitute the new generation of infected individuals who are naïve and dependent on others for care, but also require specialized intervention strategies owing to the unique nature of the infection. Furthermore, we have tried to emphasize the role of a prejudiced patriarchal society in sustaining the epidemic.

In the course of our study, it was observed that the proportion of adolescent boys seeking the services of the ICTC was more as compared to that of girls. The low turnout of girls could be due to low status and restrictions imposed by backward societies upon women thus making them ignorant of sexually acquired diseases like HIV [12–15]. In terms of HIV positivity, the proportion of males was more as compared to females. Also, males who test positive rarely divulge their HIV serostatus to their partners or spouses thereby making females oblivious to the transmission risk [16].

Most of the participants who sought ICT did so voluntarily. The percentage of males was more than that of the females which again, as discussed earlier, is likely due to the neglected and backward state of females. A small proportion of adolescents were referred by the DOT centre suggesting the presence of a referral network. However, these findings are insufficient to substantiate the significance of this referral system. Although adolescents are a vulnerable population to HIV, surprisingly the ones between 10–14 years (group I) are more predisposed to HIV than the group II. A higher probability is seen in this case as many of the adolescents are HIV positive due to the lack of prophylactics during the delivery. Also, a strong association between the HIV seropositivity and the two age groups was obtained suggesting age to be an important risk factor for HIV.

While most of the adolescents were students, a considerable portion comprised of adolescents as salaried employees in different sectors. It was also seen that illiteracy was slightly higher in female adolescents. This could be due to the lack of educational opportunities for girls and the prevailing attitudes of the society during the study period.

Another important variable analyzed in our study was marital status. Our study revealed marital status to be significantly different for males and females. This could be attributed to the widespread social practice of marrying off females earlier than males [17]. Also, marriage was found to be a potential risk factor for females. Similar findings were recorded in African studies on adolescent females [18].

HTP was recorded as the most common risk behavior and varied significantly in males and females. Also, in case of HIV-positive adolescent boys, HTP was seen as the most common risk behavior. In addition to this, percentage of unmarried adolescents in this age segment exhibiting this risk behavior was worth noting. Furthermore, males in particular were observed to be the prominent population in this category. This could be ascribed to their more experimental and carefree nature. For females of the same category, 50% of these women was married and could have perhaps acquired the infection from their spouse as also seen by Vajpayee et al. [19]. For adolescent females in Africa, the risk of HIV acquisition was noted to be directly related to risk profile of their partners [18]. However, owing to the small sample size in this case, a generalized inference cannot be drawn from our analysis. A major portion of the study population, both males and females were aged between 15 years and 19 years which could imply early coital debut. This was also seen in some African nations where males are becoming infected at slightly higher ages than females but at lower rates. This is attributed to males having either younger or more similar aged female partners at coital debut [18].

While, PTCT was the third highest risk behavior with adolescents acquiring infection which could be due to the absence or nonavailability of HIV preventive therapy during pregnancy, blood transfusion was the fourth most common risk behavior. The least common risk behavior seen was that of injected drug abuse. Our analysis also revealed that the there was a strong association between HIV seropositivity and the different types of risk behaviors among males and females. Additionally, patients classified into the heterosexual promiscuous group are 2.09 times more likely to be positive than patients whose risk group is not known. Similarly, adolescents with a potential history indicative of parent to child transmission are 3.57 times more prone to HIV than others. These results were in accordance with the NACO's data which highlighted unprotected sex (87.4% heterosexual) as the major route of HIV transmission, followed by transmission from parent to child (5.4%) and use of infected blood and blood products (1.0%) [5].

The Posttest session is an important aspect of counseling wherein the test results were disclosed to the participants. The positive cases were first counseled individually and then referred to the ART clinic where they were registered and sent for CD4 count estimation free of charge. Participants both HIV seropositive and negative were counseled. Key issues that were discussed with positive patients mainly included awareness about HIV and AIDS, the necessary precautions that should be taken, importance of healthy lifestyle, and adherence to their drug regimen.

Adolescents often encounter difficulties of a more personal nature, such as, dealing with their sexuality and peer pressure [20]. Thus, they are gullible and ready to experiment making them more vulnerable to HIV. Also, issues like sex and sexual behavior are still tabooed subjects for discussion between parents and children and even in a formal set-up between teachers and students in many parts of India. Hence, adolescents are likely to have more misconceptions and be misinformed, and in the long run, pose risk of HIV/AIDS [21]. Therefore, topics, such as, safe sexual practices, premarital issues and test of spouse or partner were stressed upon when advising sexually active adolescents. Furthermore, since HIV is generally associated with unsafe sex practices and premarital sex in the Indian society; it is considered a taboo and thus the HIV-positive adolescents face discrimination at different levels: medically, professionally, academically, and even socially. Patients complained of discriminatory behavior meted out to them by their family members. Such cases needed Counseling for the family members as well. In case of adolescent IDUs, there is a double burden of stigma of addiction and HIV infection [17]. Some of the patients were hesitant and unresponsive to certain questions possibly fearing discrimination due to their HIV serostatus which thereby rendered the ICTC ineffective in their cases. Also those who were among the high-risk behavior group but received a negative result were advised to adopt safe practices and a healthy lifestyle. This is important to avoid any further exposure to the infection. Its importance and effectiveness were validated in a study from Pune which showed that ongoing confidential Counseling and testing were positively associated with risk reduction behavior among men [22].

Although various intervention and preventive strategies against HIV have been formulated for majority of the HIV seropositive population, a more ‘adolescent-centric' approach is necessary to combat such cases. Public health policy should recognize adolescents as a separate section, design and implement prevention strategies keeping in mind their vulnerability and sensibilities for greater effectiveness of these programs.

Also awareness regarding HIV must increase among them. This could be achieved through incorporation of information regarding HIV, its transmission, and prevention into their schools' curricula. This would help in transmission of accurate information at the grass root level and would thus aid us in reducing the misconceptions and myths surrounding HIV. Furthermore, in the case of adolescent girls, orientation regarding risk of teenage pregnancy must also be discussed.

Moreover, in some western countries, Protection Motivation Theory (PMT), a social cognitive theory, has been developed and widely used to guide intervention development regarding multiple health threats, including HIV/AIDS [23]. Although its appropriateness has only been assessed in a limited number of oriental countries, such as, China, Vietnam, and the Bahamas, its applicability to the Indian scenario can be evaluated.

There were few limitations in our study. Information such as, the reason for visiting the ICTC, risk intentions, their awareness levels regarding HIV, if recorded, could have provided better insights for in-depth analysis. Additionally, owing to the small sample size, the current analysis could be considered as a pilot study that describes the profile of adolescents living with HIV in India. Therefore, the findings of this retrospective study must be followed by more elaborately designed prospective studies to provide conclusive comments on the current scenario of adolescents living with HIV in north India.

In conclusion, the present analysis has focused on the lesser known adolescent section of the population attending the ICTC in north India and draws attention to the association of HIV serostatus with variations in demographic and psychosocial responses. Not only do these findings highlight the pivotal role of ICTCs as intervention strategy, they also paint a picture representative of the current scenario of adolescents living with HIV, attending a tertiary health care centre.

## Figures and Tables

**Table 1:** Sociodemographic distribution of study population 2005–May, 2011.

Category	Males	Females
Age:		
10–14 years	132 (23.16%)	101 (30.06%)
15–19 years	438 (76.84%)	235 (69.94%)
Education:		
Illiterate	46 (7.59%)	38 (10.61%)
Primary	238 (39.27%)	146 (40.78%)
Secondary	274 (45.21%)	143 (39.94%)
Degree	48 (7.92%)	31 (8.66%)
Marital status:		
Married	22 (3.63%)	31 (8.68%)
Unmarried	584 (96.37%)	326 (91.32%)
Risk behavior:		
Heterosexual promiscuous	281 (45.77%)	149 (41.50%)
Homosexual promiscuous	3 (0.49%)	0 (0%)
Parent-to-child-transmission	94 (15.31%)	47 (13.09%)
Blood transfusion	16 (2.61%)	5 (1.39%)
Injected drug abuse	5 (0.81%)	0 (0%)
Needle prick	0 (0%)	11 (3.06%)
Others	215 (35.02%)	147 (40.97%)
HIV Status		
HIV positive	57 (9.27%)	27 (7.46%)
HIV negative	558 (90.73%)	335 (92.54%)

## References

[1] World Health Organization 10 Facts on adolescent health. http://www.who.int/features/factfiles/adolescent_health/facts/en/index.html.

[2] World Health Organization Adolescents in India: a profile. http://www.whoindia.org/LinkFiles/Adolescent_Health_and_Development_(AHD)_UNFPA_Country_Report.pdf.

[3] World Health Organization Highlights. Child and Adolescent Health and Development. Progress Report. http://whqlibdoc.who.int/publications/2010/9789241599368_eng.pdf.

[4] World Health Organization Preventing HIV/AIDS in young people—a systematic review of the evidence from developing countries. http://whqlibdoc.who.int/trs/WHO_TRS_938_eng.pdf.

[5] National AIDS Control Organization Annual Report. http://www.nacoonline.org/upload/REPORTS/NACO%20Annual%20Report%202010-11.pdf.

[6] Goparaju L Unplanned, unsafe: male students’ sexual behavior.

[7] Duong CT, Nguyen TH, Hoang TTH (2008). Sexual risk and bridging behaviors among young people in Hai Phong, Vietnam. *AIDS and Behavior*.

[8] Adimora AA, Schoenbach VJ, Doherty IA (2006). HIV and African Americans in the Southern United States: sexual networks and social context. *Sexually Transmitted Diseases*.

[9] National AIDS Control Organization Annual Report. http://nacoonline.org/upload/Publication/Annual_Report_NACO_2008-09.pdf.

[10] Quinn TC, Overbaugh J (2005). HIV/AIDS in women: an expanding epidemic. *Science*.

[11] United Nations Programme on HIV/AIDS UNGASS India. Country Progress Report. http://www.unaids.org/en/dataanalysis/monitoringcountryprogress/progressreports/2010countries/india_2010_country_progress_report_en.pdf.

[12] United Nations Programme against HIV/AIDS Gender is a crucial issue in the fight against HIV. UN development fund for women. http://www.thebody.com/content/world/art636.html.

[13] Gilbert L, Walker L (2002). Treading the path of least resistance: HIV/AIDS and social inequalities—a South African case study. *Social Science and Medicine*.

[14] Weiss E, Whelan D, Gupta GR (2000). Gender, sexuality and HIV: making a difference in the lives of young women in developing countries. *Sexual and Relationship Therapy*.

[15] Munseri PJ, Bakari M, Pallangyo K, Sandstrom E (2010). Tuberculosis in HIV voluntary counselling and testing centres in Dar es Salaam, Tanzania. *Scandinavian Journal of Infectious Diseases*.

[16] Chheng P, Tamhane A, Natpratan C (2008). Pulmonary tuberculosis among patients visiting a voluntary confidential counseling and testing center, Cambodia. *International Journal of Tuberculosis and Lung Disease*.

[17] Mawar N, Sahay S, Pandit A, Muhajan U (2005). The third phase of HIV pandemic: social consequences of HIV/AIDS stigma & discrimination & future needs. *Indian Journal of Medical Research*.

[18] Pettifor AE, Rees HV, Kleinschmidt I (2005). Young people’s sexual health in South Africa: HIV prevalence and sexual behaviors from a nationally representative household survey. *AIDS*.

[19] Vajpayee M, Mojumdar K, Raina M, Mishra S, Sreenivas V (2009). HIV voluntary counseling and testing: an experience from India. *AIDS Care*.

[20] Shisana O, Connolly C, Rehle TM, Mehtar S, Dana P (2008). HIV risk exposure among South African children in public health facilities. *AIDS Care*.

[21] Moore AR, Williamson DA (2003). Problems with HIV/AIDS prevention, care and treatment in Togo, West Africa: professional caregivers’ perspectives. *AIDS Care*.

[22] Sullivan PS, Lansky A, Drake A (2004). Failure to return for HIV test results among persons at high risk for HIV infection: results from a multistate interview project. *Journal of Acquired Immune Deficiency Syndromes*.

[23] Gong J, Saxena V, Mathur A (2010). HIV risk and prevention behaviours, intentions, perceptions and knowledge among youth in Goa, India. *International Journal of STD and AIDS*.

